# The Role of Health Literacy in Postpartum Weight, Diet, and Physical Activity

**DOI:** 10.3390/jcm9082463

**Published:** 2020-08-01

**Authors:** Rhonda Garad, Crystal McPhee, Tze Lin Chai, Lisa Moran, Sharleen O’Reilly, Siew Lim

**Affiliations:** 1 Monash Centre for Health Research and Implementation, Monash Public Health and Preventive Medicine, Monash University, and Monash Health, Melbourne 3168, Victoria, Australia; tcha0025@monash.edu (T.L.C.); Lisa.Moran@monash.edu (L.M.); siew.lm@monash.edu (S.L.); 2 Health Systems Improvement Unit, Centre for Population Health Research, School of Health and Social Development, Deakin University, Melbourne 3125, Victoria, Australia; crystal.mcphee@gmail.com; 3 Institute of Food and Health, School of Agriculture and Food Science, University College Dublin, Belfield, D04 V1W8 Dublin, Ireland; sharleen.oreilly@ucd.ie

**Keywords:** postpartum, health literacy, weight, diet, physical activity, prevention, women

## Abstract

Background: Postpartum weight retention is a significant contributor to obesity in women, adverse perinatal events in subsequent pregnancies, and chronic disease risk. Health literacy is known to impact health behaviors. The study aimed to identify the health literacy domains utilized in postpartum weight management interventions and to determine their impact on weight, diet and physical activity in postpartum women. Methods: We searched MEDLINE, CINAHL, EMBASE, PSYCINFO, and EBM databases. We included random control trials of lifestyle intervention in postpartum women (within two years post-delivery) published up to 3 May 2019. Subgroup analyses were performed to determine the effect of health literacy domains on outcomes. Results: Out of 5000 studies, 33 studies (*n* = 3905) were included in the systematic review and meta-analysis. The health literacy domain self-care (skills and knowledge) was associated with a significant reduction in body weight (mean difference (MD) −2.46 kg; 95% confidence interval (CI) from −3.65 to −1.27) and increase in physical activity (standardized mean difference (SMD) 0.61; 95% CI 0.20 to 1.02). No other health literacy domain was associated with significant outcomes in weight, energy intake, or physical activity. Conclusions: Health literacy skills such as knowledge of self-care are effective in improving weight and in increasing physical activity in postpartum women. The efficacy of other health domains was not supported.

## 1. Introduction

The postpartum period (from birth to two years) significantly contributes to weight gain and obesity in women, including the high rate of weight gain in women aged 18–30 years [1,2]. Weight retention in the postpartum period carries significant risks to maternal and child health. For women of reproductive age, excessive weight gain carries additional risks to mothers and the offspring with those gaining 3 or more Body Mass Index (BMI) units between pregnancies being at an increased risk of preeclampsia, gestational diabetes, caesarean delivery, and stillbirth in subsequent pregnancies [3]. Maternal obesity also contributes to long-term obesity and chronic disease risks in the offspring, including cardiovascular disease, diabetes, stroke, and asthma [4]. The postpartum period is, therefore, a critical window for weight management and the prevention of weight-related comorbidities of both the mother and offspring.

Health literacy is a 21st century action-oriented approach to public health, health promotion, education, and health care [5], with its importance as a determinant of health equity becoming increasingly evident. Health literacy is a multi-dimensional concept that is described by the World Health Organization as “the cognitive and social skills which determine the motivation and ability of individuals to gain access to, understand and use information in ways which promote and maintain good health” [6]. It is a determinant of health outcomes and strongly linked to empowerment and self-efficacy [7,8]. Decreased health literacy is associated with similar health outcomes as diet-related chronic disease, such as higher hospitalization rates, poorer overall health status, and higher mortality rates [9]. Health literacy has also been associated with obesity [10], poorer dietary intake [11], and reduced physical activity [12], and it may have a mediating role in the relationship between chronic conditions and health outcomes through health behaviors. Health literacy is context-dependent [13], with individual levels increasing or decreasing according to life changes such as the onset of illness or entering a new life phase [8]. Furthermore, interventions to improve health literacy developed for one population many not be effective in another.

To date, health literacy research has mainly focused on individual capacity [14], clinical assessment [15], and health system responsiveness [16], with little attention paid to the evaluation of interventions. The subsequent development of health literacy concepts has broadened the scope to address community and organization level health literacy [17]. The Health Literacy Questionnaire (HLQ) is an empirically devised, multi-faceted assessment tool that has been validated across diverse populations and within a range of contexts [16,18,19].

**Figure 1:**
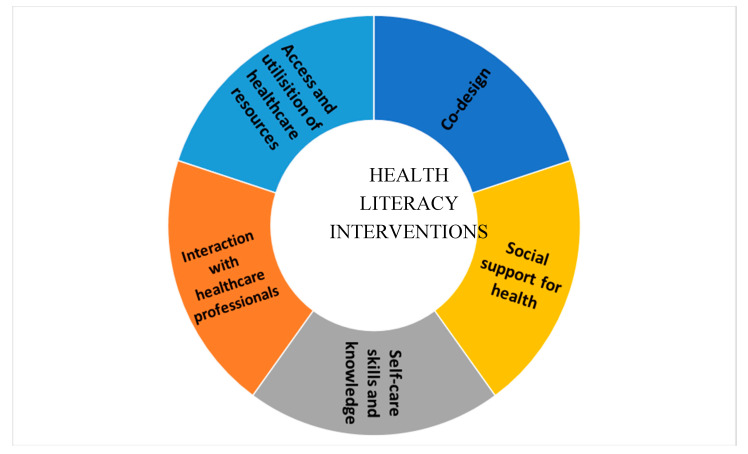
Health Literacy Evaluation Framework for Interventions.

Building on the concepts from the HLQ targeting individuals [14] that work to address health literacy in the community setting [17], we developed a health literacy framework to evaluate lifestyle interventions in the community. The Health Literacy Evaluation Framework (HLEF) for Interventions (Figure 1) is comprised of five domains: (1) interaction with healthcare professionals, (2) access and utilization of health resources, (3) self-care (skills and knowledge), (4) social support or enabler, and (5) participation in the co-design of interventions.

Postpartum women face particular challenges such as new knowledge and skill acquisition, the demands of a new born, meeting the needs of the family/community, changes in priorities, and decreased social support, all of which may impact the health literacy of postpartum women [20]. Currently, there is a lack of literature on effective health literacy domains for weight, diet, and physical activity in postpartum women.

This study aimed to identify the health literacy domains utilized in postpartum weight management interventions and to determine their impact on weight, diet, and physical activity in postpartum women. This study found that health literacy skills such as knowledge of self-care are effective in improving weight, diet, and physical activity in postpartum women. The efficacy of other health domains was not supported.

## 2. Experimental Section

### 2.1. Evidence Acquisition

This study is a secondary analysis of a systematic review that aimed to determine the effect of contextual factors on the effectiveness of lifestyle interventions in postpartum women [21].

In the current analysis, health literacy domains within lifestyle intervention trials in postpartum women were identified, and a meta-analysis to explore the relationships between each health literacy domain and body weight, energy intake, and physical activity outcomes was conducted (See Table 1).

The Preferred Reporting Items for Systematic Reviews and Meta-Analyses (PRISMA) statement for reporting systematic reviews and meta-analyses was used [23], and this systematic review was prospectively registered at PROSPERO (CRD42018086206).

### 2.2. Data Sources and Searches

Relevant studies were identified from the following databases: MEDLINE, EMBASE, PubMed PSYCINFO, CINAHL, Cochrane Database of Systematic Reviews, American College of Physicians Journal Club, Database of Abstracts of Reviews of Effects, Cochrane Central Register of Controlled Trials, Cochrane Pregnancy and Childbirth Group Trials Register, Cochrane Methodology Register, Health Technology Assessment, and the National Health Service (NHS) Economic Evaluation Database. All articles published before 3 May 2019 were included for further consideration. There was no language restriction, and translations were sought where possible. An example of the search strategies is as shown in Table S1.

### 2.3. Study Selection

The inclusion and exclusion criteria are as shown in Table S2. Randomized controlled trials involving lifestyle modification (diet, physical activity, or behavioral, e.g., stress management interventions) in postpartum women (within 2 years of childbirth) with control groups delivering minimal intervention (no more than a single session at baseline) were included. No restriction on the medical history of the participants was applied, and studies among postpartum women with depression, type 2 diabetes, and a history of gestational diabetes were considered. There was also no restriction on the baseline or pre-pregnancy BMI of participants, so postpartum women with healthy, unhealthy BMIs were considered. Studies without lifestyle modifications relevant to body weight were excluded, e.g., allergen avoidance studies. Studies commencing recruitment or implementation during pregnancy were excluded, except if a separate intervention group commencing in the postpartum period could be independently assessed. Studies that did not report any relevant outcomes in terms of body weight, BMI, energy intake, or physical activity were excluded. Editorials, reviews, conference abstracts, letters, commentaries, uncontrolled trials, non-randomized trials, and study protocols were excluded to ensure that only the highest level of evidence was included in this systematic review and meta-analysis [24]. Two reviewers from a pool of three independently assessed the eligibility of the articles. Discrepancies were resolved by consensus and arbitration.

### 2.4. Data Extraction

The general characteristics of the study (country, sample size, education (highest level) ethnicity, socioeconomic status (highest income), participants (sampling frame, inclusion and exclusion criteria, postpartum stage), and outcomes (body weight, BMI, energy intake, and physical activity) were extracted from the included studies (Table 2). The country of study was categorized as developed or developing countries according to the United Nations classification. Interventions were coded for the presence or absence of health literacy domains within the HLEF for Interventions. The domains were (1) interaction with healthcare professionals, (2) access and utilization of health resources, (3) self-care (skills and knowledge), (4) social support or enabler, and (5) participation in the co-design of interventions. Domains 1–4 were derived from the Health Literacy Questionnaire [14] targeted at individuals, whilst domain 5 was added to capture the development of health literacy interventions targeted at populations or groups as previously described [17]. The definitions of the domains is as shown in Table S3. The general characteristics of the study were independently extracted by two reviewers (Eliza Tassone and Christina Cheng). Health literacy domains were coded independently by two reviewers (Rhonda Garad and Crystal McPhee), and discrepancies were resolved by consensus (Rhonda Garad, Crystal McPhee and Siew Lim).

### 2.5. Quality Assessment

The quality of the included studies was critically appraised using the Revised Cochrane Risk of Bias Tool for Randomized Trials (RoB 2.0) for parallel group trials, which assessed bias associated with starting and adhering to intervention [61] (Table S4). This tool assessed bias associated with the randomization process, deviations from the intended protocol, missing outcome data, measurement of the outcome, and selective reporting. One reviewer (X.L.) appraised all the articles, while another (C.C.) independently appraised 10% of randomly selected studies. An inter-rater agreement of 83% was achieved (Cohen’s kappa = 0.67). Discrepancies were resolved by consensus.

### 2.6. Data Synthesis and Analysis

Weight and energy intake outcomes were expressed as mean differences (MDs), while physical activity outcomes were expressed as standardized mean differences (SMDs), both with 95% confidence intervals (CIs). Due to heterogeneity, outcomes were combined in the meta-analysis with the inverse variance random-effects model (DerSimionian and Laird method) [62]. Between-study heterogeneity was assessed using the chi-square test (*p* < 0.1 was considered statistically significant). Inconsistency between the studies was assessed using the I^2^ test (I^2^ < 25% was considered low heterogeneity, and I^2^ > 50% was considered substantial heterogeneity). Possible sources of heterogeneity based on the inclusion or exclusion of each health literacy domain were explored in univariate meta-regression for weight, diet, and physical activity outcomes. Publication bias was explored using funnel plots and Egger’s tests. Data analysis was performed using RStudio 1.1.463 (Free Software Foundation, Inc., Boston, MD, USA).

### 2.7. Evidence Synthesis

#### 2.7.1. Identification of Studies

**Figure 2:**
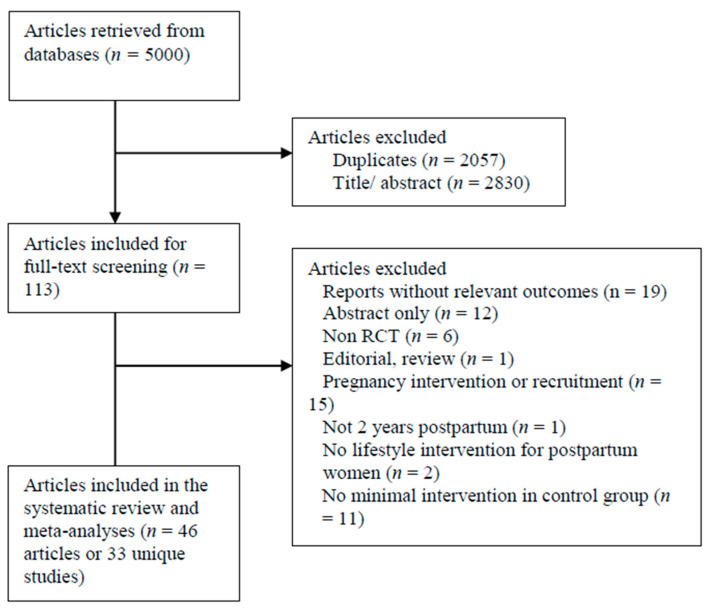
Preferred Reporting Items for Systematic Reviews and Meta-Analyses (PRISMA) procedural flow chart of search and identification process.

A total of 5000 articles were identified, as shown in Figure 2. After initial exclusion based on titles and abstracts, 113 full-texts were screened using the selection criteria. Of these, 46 articles met the inclusion criteria. The reasons for exclusions were no minimal intervention in the control group, no relevant outcomes, recruitment during pregnancy, more than two years postpartum, or being a publication type that did not meet the inclusion criteria. Due to reports on secondary analyses of original interventions, these 46 articles represented 33 unique studies (*n* = 3905 participants) that were included in this systematic review and meta-analyses.

#### 2.7.2. Study Characteristics

The characteristics of the included studies are shown in Table 1 and Table S5. The smallest studies had 24 participants [31,48], while the largest study had 542 participants [43]. All included studies were available in the English language. The majority of the studies were conducted in developed countries (27/33 studies). Most (20/33) studies investigated a combination of diet and physical activity interventions, while 12 studies investigated the sole effects of physical activity, and one study evaluated the effects of a diet-only intervention (Table S5). Recruitment targeted postpartum women from 3 weeks [48] to 18 months [43]. In about one-third of the studies (10/33), more than half the participants had tertiary education [26,36,43,49,52,53,54,55,57,59]. Twenty-two studies reported the ethnicity of the participants, of which 13 reported participants being mostly Caucasians (Table 1).

#### 2.7.3. Quality Assessment

The quality of the studies assessed through the risk of bias is as shown in Table S4. Most studies had a high overall risk of bias, mostly due to bias resulting from deviations from intended interventions. The risk of bias from missing outcome data was reduced through the intention-to-treat analysis reported in ten studies [29,33,36,37,41,42,43,52,53,56,63]. Two studies [38,44,45,46] reported higher drop-out rates in the intervention group, which could have been related to the true outcome, and these studies consequently received a rating of high risk of bias from missing outcome data. One-third of the studies reduced detection bias through the use of objective measures such as step counts, thus receiving a low risk of bias on the domain ‘measurement of outcome.’ None of the studies were deemed at risk of selective reporting. Funnel plots suggested a low risk of publication bias due to largely symmetrical plots for body weight and energy intake (Figure S1). However, funnel plots suggested potential publication bias for physical activity outcomes, with the possibility of small studies reporting negative outcomes such as an increase in energy intake or a decrease in physical activity not being published (Figure S1).

## 3. Results

The health literacy domains of individual studies are as shown in Table S6. No studies reported interaction with healthcare professionals, three studies reported access and utilization of health resources [29,38,53], all studies reported self-care (skills and knowledge), eleven studies reported social support or enabler (Table 3), and one study reported participation in decision-making or co-design [41].

A meta-analysis found that lifestyle modification in postpartum women was associated with significant reductions in body weight (MD: −2.46 kg; 95% CI: from −3.65 to −1.27; 25 studies; 1945 participants; I2 = 79%) and physical activity (SMD: 0.61; 95% CI: from 0.20 to 1.02; 24 studies; 2138 participants; I2 = 86%) but not for energy intake (MD: −605 KJ/day; 95% CI: from −1530 to 320; 12 studies; 1123 participants; I2 = 82%) (Figure S1). As all studies reported self-care (skills and knowledge), the meta-analysis findings also reflected the findings of this health literacy domain. Heterogeneity were further explored through meta-regression on the access and utilization of health resources and social support or enabler. These health literacy domains were not significantly associated with outcomes in weight, energy intake, or physical activity (Table S6).

## 4. Discussion

Using a comprehensive health literacy definition within the HLEF for Interventions, we evaluated the relationships between each health literacy domain and the outcomes of body weight, energy intake, and physical activity in lifestyle interventions for postpartum women. Using a health literacy lens to the health issue of postpartum weight management is critical, because current interventions are only focused on a narrow component of health literacy within current design thinking. The inclusion of the HLEF can improve the health literacy of the participant through a co-design approach that enables them to address weight management with the supports they have available to them (community, social, and health care practitioner) in their own environments.

All lifestyle intervention trials involved health literacy skills such as self-care (skills and knowledge) in diet or physical activity, but limited studies reported strategies that enable women to make use of existing resources for health and well-being or to increase social support for health. There was a lack of studies addressing health literacy domains such as engaging healthcare professionals or co-developing interventions or programs. Interventions that increase skill and knowledge were associated with significant improvements in weight loss and increases in physical activity. No other health literacy domains had a significant benefit on body weight, energy intake, or physical activity outcomes in postpartum women.

By default and definition, all lifestyle interventions reported some form of instruction or information on diet or exercise, thus consistent with domain 3: self-care (skills and knowledge) of the HLEF for Interventions. As such, all studies in this review reported the self-care (skills and knowledge) domain of the framework. We found that these interventions significantly improved outcomes in body weight and physical activity in postpartum women. This is consistent with previous systematic reviews and meta-analyses of lifestyle interventions in postpartum women [64,65]. In these previous systematic reviews and meta-analyses, health literacy was not assessed, but it is likely that the targeted health literacy skills were within domain 3 of the HLEF. Similar association between these domains and anthropometric and dietary outcomes were also previously reported in other populations [11,66].

None of the lifestyle interventions in postpartum women were aimed at empowering women to engage with healthcare professionals to manage their weight or lifestyle behaviors such as diet or physical activity. This is despite engagement with healthcare professionals having been identified as one of the most important aspects of effective weight management in postpartum women [21]. This is a significant gap in research and practice. An example of an intervention targeting the health literacy domain of healthcare engagement is a patient-held clinical tool such as the Question Prompt List (QPL). A QPL has been developed for women with polycystic ovary syndrome (PCOS). It is an evidence-based selection of questions that were co-designed with women with the chronic condition polycystic ovary disease [67]. The questions cover the range of inquiries deemed important by women with this condition. The QPL increases health literacy by optimizing interactions with health professionals, increasing awareness of individual knowledge gaps, and facilitating information seeking. Finally, the QPL increases confidence to initiate help-seeking and to effectively interact with healthcare professionals. The QPL builds on the health literacy skills and facilitates the progression towards higher level and more comprehensive health literacy skills such as making decisions with health professionals on health. Failure to expand individual health literacy skills is a missed opportunity to empower, foster independence, and achieve sustained behavior change [68].

Very few of the lifestyle interventions in postpartum women sought to increase knowledge of existing services and to support to manage their weight. One example in this review was a study that provided women with information and opportunities to access walking paths suitable for prams [69]. Physical and environmental barriers such as not having opportunities for physical activity have been cited as significant barriers by women of reproductive age [70,71]. The provision of information on community resources that facilitate healthy diet and physical activity that addresses the specific needs of postpartum women may have been beneficial. However, our current meta-analysis found that this health literacy domain, by itself, is insufficient to produce significant weight and behavioral changes within the context of an intervention trial. However, this finding may have been limited by the low number of studies reporting these health literacy strategies.

Few studies targeted the women’s social support networks to assist in the management of weight or lifestyle behaviors such as diet and physical activity. In contrast to the findings of one observational study that found that social support predicted increased fruit and vegetable intake [71], the current analysis did not find a significant benefit for this health literacy domain by itself. A single study [72] directly involved someone from the participant’s social network, and none involved the participant’s partner. This may explain the limited effect seen in the current meta-analyses for this health literacy domain. The demands of a newborn, other family needs, changes in priorities, and a lack of social support can all reduce a postpartum women’s ability to engage in lifestyle modification activities [16,73,74]. Considering the crucial role of these family relations in determining lifestyle behaviors, reorienting these or other social relationships to support postpartum health would be an important step in engaging women [20]. Future interventions should explore strategies to strengthen the existing social support for postpartum mother’s health [20].

Only one study in this review consulted postpartum women in intervention development [75]. Considering the overall low reach and engagement of postpartum women in the interventions [15,65], researchers should consider involving postpartum women in the co-design of interventions to increase both relevance and fit-for-purpose interventions that are implementable in local contexts. Improving reach and engagement is particularly important to optimize health impact at the population level [76]. The partnership and involvement of the end-user have been identified as vital strategies to engage hard-to-reach populations [20,77], as they influence retention, penetration, and participation. Co-creation models such as value co-creation, experience-based co-design, technology co-design, and community-based participatory research has been shown to improve the effectiveness of interventions in other populations [78]. Future interventions in postpartum women should involve these women in the development of the lifestyle intervention.

### Limitations

Firstly, health literacy information was not typically reported within interventions, even if health literacy approaches were used. As a result of this deficit in reporting, researchers trained in using health literacy approaches and lifestyle intervention delivery methods coded each study using a health literacy framework and a rigorous quality appraisal approach. Secondly, the comprehensive health literacy concept came from chronic disease management [14], and it is possible that certain domains, such as access and utilization of health resources, may have less influence on disease prevention outcomes such as weight management or weight gain prevention. Thirdly, the paucity of studies limited our ability to assess the effects of other domains on the overall outcomes of interest.

## 5. Conclusions

This systematic review and meta-analysis of lifestyle interventions in postpartum women summarized the evidence from a comprehensively-defined health literacy perspective to inform research and practice. The findings suggest that self-care (skills and knowledge) in the HLEF is effective in improving weight, diet, and physical activity for postpartum women. Evidence to support the efficacy of other health literacy domains such as utilizing existing resources for health and well-being, increasing social support for health, engaging healthcare professionals, or co-developing interventions or programs was limited.

The lack of evidence on the effect of health literacy domains such as engaging with healthcare professionals and the co-development of interventions on postpartum weight, diet, and physical activity and behaviors demonstrated that research on other health literacy domains in addition to self-care (skills and knowledge) is needed. Effective intervention designs that increase health literacy are likely to improve the reach, engagement, and long-term effectiveness of interventions on postpartum weight and lifestyle behaviors such as diet and physical activity.

This paper makes an important contribution to the evidence-base on role of health literacy in postpartum weight gain prevention and management, leading to improved perinatal outcomes and risk reduction for maternal and child chronic disease onset. Obesity is an unsolved crisis that is generating long term distress and disabilities, reducing human capital, and increasing disease burdens and healthcare costs globally. The postpartum period provides a critical window of opportunity to impact maternal and child weight gain trajectory. Patients have identified that all domains within the HLQ as important; therefore, public health researchers should consider the inclusion of all domains of health literacy in study design, and they should incorporate co-design processes to ensure high utility to end-users. In addition, health professionals should consider all health literacy domains when providing health advice to patients, although the authors do acknowledge that at this point in time, few resources are available that use an evidence-based approach to health literacy to facilitate this, thus highlighting the need for further health research in this area.

## Figures and Tables

**Table 1:** Definitions of health literacy lifestyle intervention domains.

Domain Name	Included Domains from HLQ	Example
Interacting with healthcare professionals (HP) *	D6—Ability to actively engage with healthcare providers (AE)	Example: a tool or intervention (practical) that augments aids communication with HP. Permission to ask questions. Understanding roles of HPs. Language to ask questions. Understanding their own needs. Reluctance of HPs to discuss weight/lifestyle.
Access and utilization of health care	D7—Navigating the healthcare system (NHS)	Example: provide advice or resource list on where to seek help to manage weight and lifestyle—can include community resources Access and utilization of health resources. (e.g., knowledge and limitation around having a child). Teach how to use existing resources in the community.
Self-care (skills and knowledge)	D3—Actively managing my health (AMH) D2—Having sufficient information to manage my health (HSI) D5—Appraisal of health information (CA) D8—Ability to find good health information (FHI) D9—Understand health information well enough to know what to do (UHI)	Example: goal setting, self-monitoring, meal or exercise plans, relapse coping, stress coping, cognitive behavioral therapy, specific time, intensity, and duration of exercise. Example: provide instruction on how to perform behavior/diet/exercise, demonstration, and onsite sessions.
Social support or enabler	D4—Social support for health (SS)	Example: social circle/peer natural social circle/family/partners, including leveraging social relations enabling her health/purposeful interaction. Intentional/explicit/not-incidental social support as a resource to manage health. Lack of intentional social support.
Participating in health debates and decision-making	Not in HLQ	Example: Involve in the development of implementation of the intervention/co-design

HLQ: Health Literacy Questionnaire. * HP is a registered health practitioner under national law to practice a health profession or someone who holds a non-practicing registration in a health profession under national law [22].

**Table 2:** Description of cohorts reported in the included studies.

Study	Country	Education (Highest Level)	* Ethnicity/ Nationality	SES (Highest Income If Reported)
Berry et al., 2015 [25]	USA	13% university graduates	77% African-American; 23% Non-Hispanic White	28% $20,000–$39,999 per year.
Bertz et al., 2015 [26]	Sweden	69–80% had > 3 y beyond high school	n/a	n/a
Colleran et al., 2012 [27]	USA	All participants except one had at least a university education	85% White, non-Hispanic; 11% African-American; 4% Hispanic	n/a
Craigie et al., 2011 [28]	UK	28–35% degree attained	93–96% Caucasian	13% > £40,000
Daley et al., 2015 [29]	UK	n/a	57–68% White	6–9% Index of Multiple Deprivation (IMD) quartile 1 (least deprived)
Davenport, 2011 [30]	Canada	n/a	85–90% Caucasian	n/a
deRosset et al., 2013 [31]	USA	42% completed high school	100% Hispanic	21% had household income from $20,000–$39,999
Dritsa et al., 2009 [32]	Canada	15–16 mean years of education	n/a	Mean 4.9–5.25 (4 = $30,000–$40,000; 5 = $40,000–$50,000)
Fjeldsoe et al., 2010 [33]	Australia	16–17% had the highest education of year 10	2–6% identified as an Aboriginal or Torres Strait Islander	2–6% had a weekly household income < $600
Holmes et al. 2018 [34]	USA	16–17 mean years of education	Caucasian	n/a
Huang et al., 2009 [35]	Taiwan	23–28% university and above	n/a	n/a
Huseinovic et al., 2016, 2018 [36,37]	Sweden	60% > 3 y beyond high school	n/a	n/a
Keller et al., 2014 [38]	USA	n/a	100% Latina	14% household income > $30,000
Kernot et al., [39]	Australia	85% tertiary education	n/a	n/a
Khodabandeh et al., 2017 [40]	Iran	13–28% university degrees	99–100% Azeri	55–68% reported income equal to expenses
Krummel et al., 2010 [41]	USA	60% had at least a high school education	90% Caucasian	65% stay-at-home mothers
Leermakers et al.,1998 [42]	USA	12–30% graduate degree	95–98% Caucasian	n/a
Lioret et al., 2012 [43]	Australia	54% university degree or higher	79% Australian; 21% Other	n/a
Lovelady et al., 2000, 2001, 2006 [44,45,46]	USA	n/a	80–84% White; 16–19% Black	n/a
Lovelady et al., 1995 [47]	USA	16–17 mean years of education	n/a	n/a
Lovelady et al., 2009 [48]	USA	n/a	95% Non-Hispanic White; 5% Asian.	n/a
Maturi et al., 2011 [49]	Iran	44–47% diploma; 41–48% university education	n/a	22–24% employed
McCrory et al., 1999 [50]	USA	16–17 mean years of education	77–82% Non-Hispanic White; 9–14% Hispanic; 0–13% Black; 0–9% Asian	n/a
McIntyre et al., 2012 [51]	Australia	60–62% > high school	n/a	n/a
Nicklas et al., 2014 [52]	USA	20–28% some university; 56–60% university graduate	51–64% White; 25–36% African American; 11–13% Asian; 15–25% Hispanic or Latina	29–38% Low-income
Ostbye et al., 2009 [53]	USA	24–25% some university; 54–56% university or more	52–53% White; 45% Black; 2–3% Asian/Other	42–43% > $60,000
O’Toole et al., 2003 [54]	USA	75% university graduates	98% Caucasian; 3% African American	43% full-time home-makers
Parsa et al., 2017 [55]	Iran	30–32% diploma; 11% associate degree; 18% bachelor	n/a	11% more than two million toman per month (1 USD = 3800 toman)
Tripette et al., 2014 [56]	Japan	n/a	100% Japanese	n/a
Wiltheiss et al., 2013 [57]	USA	20% some college or vocational, 42% college graduate, 27% graduate school	75% White; 22% black; 4% other races; 5% Hispanic	57% household income > $60,001
Youngwanichsetha et al., 2013 [58]	Thailand	31–38% Bachelor’s degree or higher	n/a	n/a
Zourladani et al., 2014 [59]	Greece	50% university graduates	100% Greek	n/a
Zilberman et al., 2018 [60]	Israel	11 mean years of education	Jewish and Bedouin	n/a

* As reported by the authors. SES: Socioeconomic status.

**Table 3:** Univariate meta-regression of lifestyle interventions in postpartum women on body weight, energy intake, and physical activity by health literacy domains.

Health Literacy Domains	β	95% Confidence Interval	*p*-Value	Adjusted R-Squared (%)
*Weight*				
Access and utilization of health resources	1.92	−2.04, 5.88	0.33	0
Social support or enabler	−0.47	−3.22, 2.29	0.73	0
*Energy intake*				
Access and utilization of health resources	0.89	−1.55, 3.34	0.44	0
Social support or enabler	1.02	−0.86, 2.90	0.25	4.9
*Physical activity*				
Access and utilization of health resources	−0.62	−2.04, 0.81	0.38	0
Social support or enabler	−0.31	−1.19, 0.56	0.47	0

β = regression coefficient; CI = confidence interval; k = number of evaluations; adjusted R^2^ = adjusted proportion of heterogeneity accounted for by moderator.

## Supplementary Materials

The following are available online at https://www.mdpi.com/2077-0383/9/8/2463/s1, Figure S1. Forest plots and funnel plots for weight, energy intake and physical activity, Table S1: Search strategies, Table S2: Inclusion and exclusion criteria of the systematic review and meta-analysis of lifestyle intervention in postpartum women, Table S3: Definition of Health Literacy Lifestyle Intervention Domains, Table S4: Risk of bias of included studies *, Table S5: Health literacy domains of lifestyle interventions in postpartum women, Table S6. Health literacy domains of lifestyle interventions in postpartum women.
