# Supplementary material for: The Role of Health Literacy in Postpartum Weight, Diet, and Physical Activity
Source: J Clin Med. 2020 Aug 1;9(8):2463. doi: 10.3390/jcm9082463 (PMC7465022; doi:10.3390/jcm9082463)
Supplement: Supplementary file 1 [file jcm-09-02463-s001.pdf]

Study	Experimental Total	Experimental Mean	Experimental SD	Control Total	Control Mean	Control SD	MD	95%-CI	Weight
Bertz 2012,2014,2015	15	-7.30	6.3000	13	-0.90	6.6000	-6.40	[-11.20; -1.60]	2.8%
Colleran 2012	14	-5.80	3.5000	13	-1.60	5.4000	-4.20	[-7.66; -0.74]	3.7%
Craigie 2011	22	-1.60	2.0000	14	0.20	2.2000	-1.80	[-3.22; -0.38]	5.2%
Daley 2015	41	75.40	16.0500	38	77.11	18.3900	-1.71	[-9.35; 5.93]	1.6%
Davenport 2011	20	-5.00	2.9000	20	-0.10	3.3000	-4.90	[-6.83; -2.97]	4.9%
Holmes 2018	20	-3.90	7.0000	25	0.70	3.9000	-4.60	[-8.03; -1.17]	3.8%
Huang 2009	64	56.67	6.1200	64	58.61	10.2300	-1.94	[-4.86; 0.98]	4.1%
Huseinovic 2016,2017	41	-6.90	7.2000	46	-4.60	6.9000	-2.30	[-5.27; 0.67]	4.1%
Keller 2014, 2015	39	71.99	10.3800	54	72.54	11.3000	-0.55	[-4.99; 3.89]	3.1%
Krummel 2010	24	1.31	5.3520	33	1.31	4.8530	0.00	[-2.71; 2.71]	4.3%
Leermakers 1998	36	-7.80	4.5000	26	-4.90	5.4000	-2.90	[-5.44; -0.36]	4.4%
Lovelady 1995	18	65.70	2.5000	15	65.40	2.1000	0.30	[-1.27; 1.87]	5.1%
Lovelady 2000, 2001, 2006	21	-4.80	1.7000	19	-0.80	2.3000	-4.00	[-5.26; -2.74]	5.3%
Lovelady 2009	10	65.10	3.5000	10	65.20	4.1000	-0.10	[-3.44; 3.24]	3.8%
Maturi 2011	32	64.70	8.0000	34	63.90	6.0000	0.80	[-2.63; 4.23]	3.8%
McCrory 1999	22	-1.60	0.5000	23	-0.20	0.6000	-1.40	[-1.72; -1.08]	5.6%
McIntyre 2012	14	0.97	3.7000	11	0.22	4.2000	0.75	[-2.40; 3.90]	4.0%
Nicklas 2014	36	-3.00	5.9110	39	1.00	5.8613	-4.00	[-6.67; -1.33]	4.3%
Ostbye 2009	214	-0.90	5.1000	207	-0.36	4.9000	-0.54	[-1.50; 0.42]	5.4%
O'Toole 2003	13	71.30	2.2000	10	84.10	4.3000	-12.80	[-15.72; -9.88]	4.1%
Tripette 2014	17	-2.20	0.9000	17	-0.50	0.7000	-1.70	[-2.24; -1.16]	5.5%
Wiltheiss 2012	131	-2.30	5.4000	145	-1.50	4.7000	-0.80	[-2.00; 0.40]	5.3%
Youngwanichsetha 2013	32	68.90	14.5000	32	72.60	17.3900	-3.70	[-11.54; 4.14]	1.6%
Zilberman 2018	60	73.11	16.1100	44	76.68	15.9100	-3.57	[-9.79; 2.65]	2.1%
Zourladani 2015	20	61.80	10.0000	17	64.20	8.5200	-2.40	[-8.37; 3.57]	2.2%
**Random effects model**	**976**			**969**			**-2.46**	**[-3.65; -1.27]**	**100.0%**

Heterogeneity: $I^2 = 79\%$, $\tau^2 = 6.1530$, $p < 0.01$

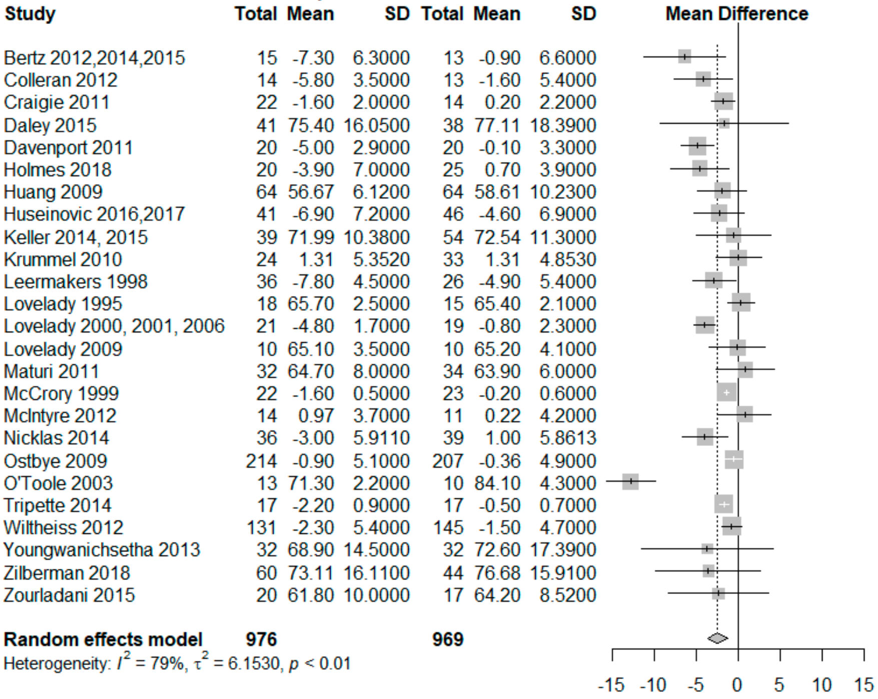

Study	Experimental Total	Experimental Mean	Experimental SD	Control Total	Control Mean	Control SD	Mean Difference	MD	95%-CI	Weight
Bertz 2012,2014,2015	15	-3.59	2.7820	13	-1.39	1.8660		-2.20	[-3.94; -0.46]	6.8%
Colleran 2012	14	-2.56	2.1799	13	-0.72	1.8200		-1.85	[-3.36; -0.34]	7.3%
Davenport 2011	20	7.90	1.5970	20	8.32	1.8640		-0.42	[-1.49; 0.66]	8.4%
Huseinovic 2016,2017	44	-2.75	3.2426	45	1.42	3.6777		-4.17	[-5.61; -2.73]	7.5%
Keller 2014, 2015	39	5.83	2.1548	54	5.42	1.6276		0.41	[-0.39; 1.22]	9.1%
Lovelady 1995	18	10.45	1.8243	15	9.22	1.3600		1.23	[0.14; 2.32]	8.4%
Lovelady 2000, 2001, 2006	19	-2.28	1.9707	16	-0.99	2.1255		-1.29	[-2.66; 0.08]	7.7%
Lovelady 2009	10	8.05	1.6539	10	7.07	1.5745		0.97	[-0.44; 2.39]	7.6%
Ostbye 2009	214	-0.87	2.2761	207	-0.70	2.2510		-0.16	[-0.60; 0.27]	9.7%
O'Toole 2003	13	6.66	0.3054	10	6.45	0.3724		0.21	[-0.07; 0.50]	9.9%
Tripette 2014	17	-0.86	2.3389	17	-0.11	1.0962		-0.75	[-1.98; 0.48]	8.1%
Wiltheiss 2012	131	7.36	2.1589	145	7.70	2.1757		-0.33	[-0.85; 0.18]	9.6%
**Random effects model**	**554**			**565**				**-0.61**	**[-1.53; 0.32]**	**100.0%**

Heterogeneity: $I^2 = 82\%$, $\tau^2 = 1.6501$, $p < 0.01$

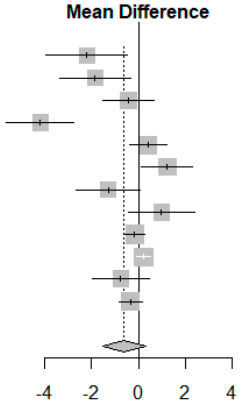

Study	Experimental Total	Experimental Mean	Experimental SD	Control Total	Control Mean	Control SD	Standardised Mean Difference	SMD	95%-CI	Weight
Berry	24	2.30	0.5000	20	1.90	0.6000		0.72	[0.10; 1.33]	4.2%
Bertz 2012,2014,2015	15	1588.00	2652.0000	13	766.00	3247.0000		0.27	[-0.48; 1.02]	3.9%
Colleran 2012	14	34.50	7.2000	13	33.80	7.2000		0.09	[-0.66; 0.85]	3.9%
Craigie 2011	22	19.00	47.0000	14	16.00	48.0000		0.06	[-0.61; 0.73]	4.1%
DeRosset	13	0.90	0.4900	11	0.70	0.5900		0.36	[-0.45; 1.17]	3.8%
Dritsa	46	124.09	96.3300	42	54.60	55.8000		0.87	[0.43; 1.30]	4.5%
Fjeldsoe	45	18.26	24.9400	43	16.36	25.5300		0.07	[-0.34; 0.49]	4.5%
Huseinovic 2016,2017	44	1053.00	2440.0000	45	394.00	2857.0000		0.25	[-0.17; 0.66]	4.5%
Keller 2014, 2015	39	6963.91	3126.5400	54	6425.04	3390.9000		0.16	[-0.25; 0.58]	4.5%
Kernot 2019	41	173.00	101.3000	40	160.00	77.4000		0.14	[-0.29; 0.58]	4.5%
Khodabandeh	105	16.00	15.2000	101	8.00	7.9000		0.65	[0.37; 0.93]	4.7%
Krummel 2010	18	705.00	2475.0000	24	308.00	2977.0000		0.14	[-0.47; 0.75]	4.2%
Lioret	178	387.72	346.2800	179	403.62	363.7900		-0.04	[-0.25; 0.16]	4.7%
Lovelady 1995	18	33.80	1.0000	15	28.90	1.1000		4.57	[3.21; 5.93]	2.8%
Lovelady 2000, 2001, 2006	21	4.50	4.9000	19	0.60	3.8000		0.87	[0.21; 1.52]	4.1%
Lovelady 2009	10	34.70	4.7434	10	34.40	4.1110		0.06	[-0.81; 0.94]	3.7%
Maturi 2011	32	11.00	34.4000	34	8.00	23.5000		0.10	[-0.38; 0.58]	4.4%
McCrory 1999	22	499.00	87.0000	23	135.00	126.0000		3.29	[2.37; 4.21]	3.6%
Ostbye 2009	214	21.80	143.6000	207	31.10	146.6000		-0.06	[-0.26; 0.13]	4.7%
O'Toole 2003	13	1981.00	72.0000	10	1902.00	79.0000		1.01	[0.13; 1.90]	3.7%
Parsa	56	17.91	3.0000	56	13.88	2.7100		1.40	[0.99; 1.81]	4.5%
Tripette 2014	17	25.90	3.3000	17	26.40	2.8000		-0.16	[-0.83; 0.51]	4.1%
Zilberman 2018	60	28.00	46.7000	44	1.00	2.3000		0.75	[0.35; 1.16]	4.5%
Zourladani 2015	20	1.96	0.3500	17	1.70	0.2600		0.82	[0.14; 1.49]	4.1%
**Random effects model**	**1087**			**1051**				**0.61**	**[0.20; 1.02]**	**100.0%**

Heterogeneity: $I^2 = 86\%$, $\tau^2 = 0.6588$, $p < 0.01$

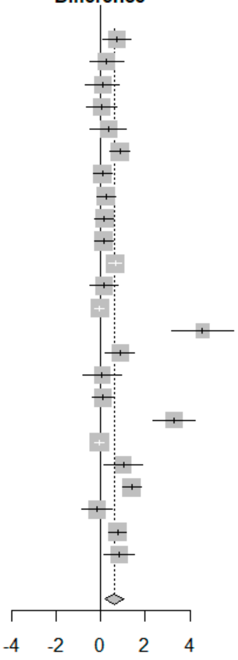

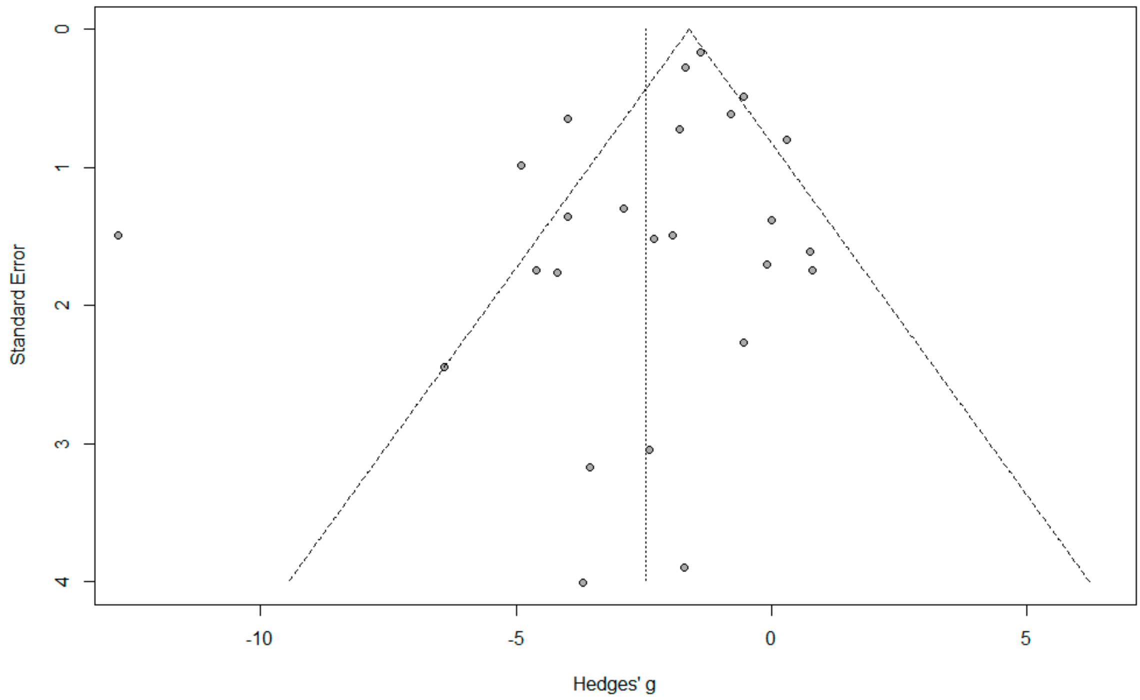

0
1
2
3
4
Standard Error
-10
-5
0
5
Hedges' g

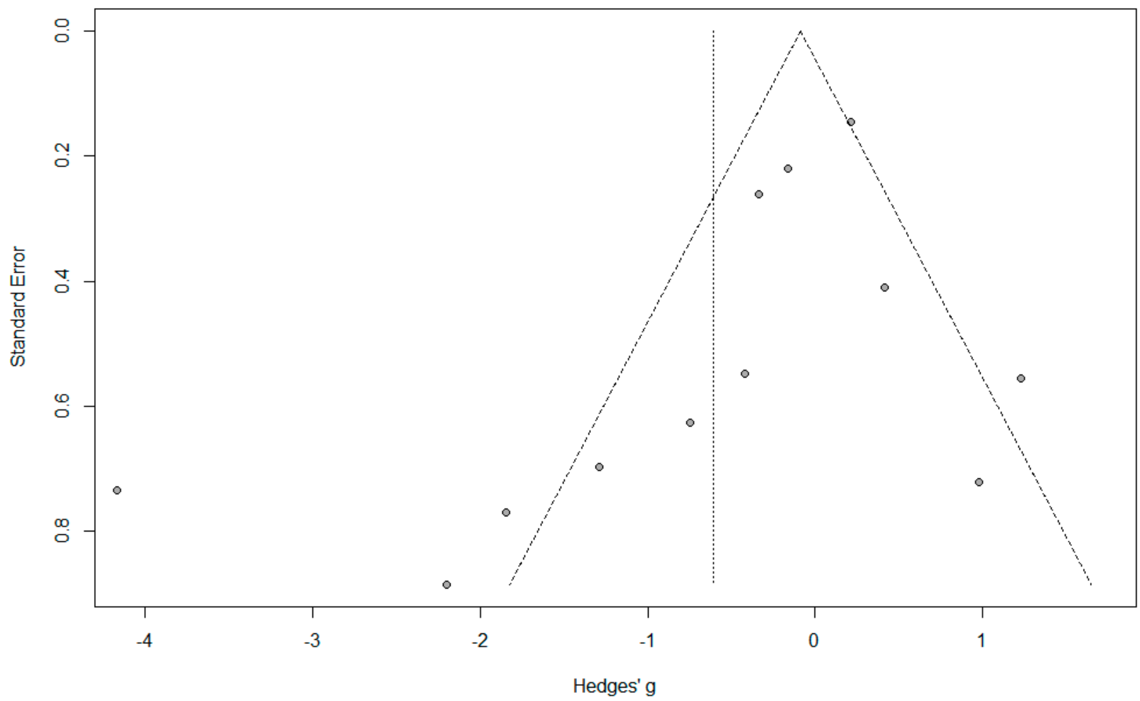

0.0
0.2
0.4
0.6
0.8
Standard Error
-4
-3
-2
-1
0
1
Hedges' g

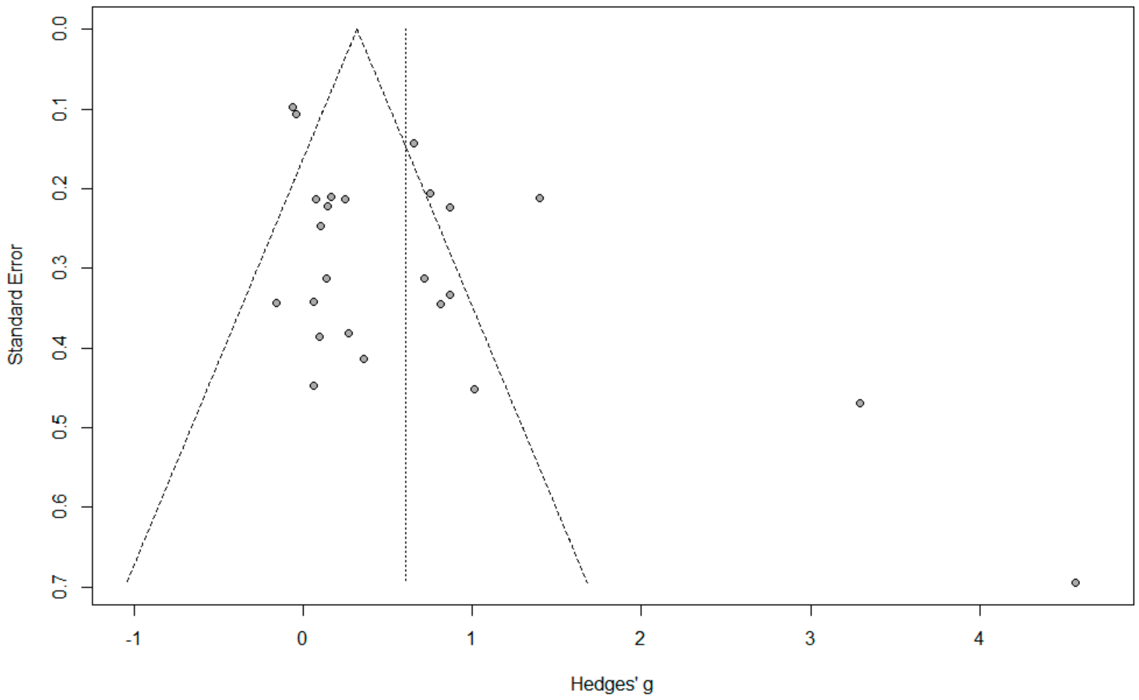

Figure S1. Forest plots and funnel plots for weight, energy intake and physical activity. (**A**) Forest plot for body weight. (**B**) Forest plot for energy intake. (**C**) Forest plot for physical activity. (**D**) Funnel plot for body weight. (**E**) Funnel plot for energy intake. (**F**) Funnel plot for physical activity.

Table S1. Search strategies.

Medline

1. (MH "Postpartum Period+")
2. TI (postpartum OR post-partum OR postnatal OR post-natal OR puerperium OR postpartal OR post-partal OR lactating OR lactation OR "nursing women" OR breastfeeding OR breast-feeding OR "after birth" OR "following pregnancy" OR postpregnancy OR "post pregnancy" OR "following childbirth" OR "after delivery" OR "post childbirth") OR AB (postpartum OR post-partum OR postnatal OR post-natal OR puerperium OR postpartal OR post-partal OR lactating OR lactation OR "nursing women" OR breastfeeding OR breast-feeding OR "after birth" OR "following pregnancy" OR postpregnancy OR "post pregnancy" OR "following childbirth" OR "after delivery" OR "post childbirth")
3. 1 or 2
4. TI diet* OR AB diet*
5. TI (life*style N2 (chang* OR intervention*)) OR AB (life*style N2 (chang* OR intervention*))
6. TI ("physic* activ*" OR exercis*) OR AB ("physic* activ*" OR exercis*)
7. 4 or 5 or 6
8. 3 and 7
9. (MH "Randomized Controlled Trial+")
10. (MH "Clinical Trial+")
11. randomi?ed controlled trial$.tw.
12. RCT.tw.
13. random allocation.tw.
14. randomly allocated.tw.
15. allocated randomly.tw.
16. (allocated adj2 random).tw.
17. or/9-16
18. 8 and 17

Limit: Humans

CINAHL

1. (MH "Postnatal Period+") OR (MH "Postnatal Care+")
2. TI (postpartum OR post-partum OR postnatal OR post-natal OR puerperium OR postpartal OR post-partal OR lactating OR lactation OR "nursing women" OR breastfeeding OR breast-feeding OR "after birth" OR "following pregnancy" OR postpregnancy OR "post pregnancy" OR "following childbirth" OR "after delivery" OR "post childbirth") OR AB (postpartum OR post-partum OR postnatal OR post-natal OR puerperium OR postpartal OR post-partal OR lactating OR lactation OR "nursing women" OR breastfeeding OR breast-feeding OR "after birth" OR "following pregnancy" OR postpregnancy OR "post pregnancy" OR "following childbirth" OR "after delivery" OR "post childbirth")

3. 1 or 2
4. TI diet* OR AB diet*
5. TI (life*style N2 (chang* OR intervention*)) OR AB (life*style N2 (chang* OR intervention*))
6. TI (“physic* activ*” OR exercis*) OR AB (“physic* activ*” OR exercis*)
7. 4 or 5 or 6
8. 3 and 7
9. Clinical trial/
10. randomized controlled trials/
11. random allocation/
12. randomi?ed controlled trial$.tw.
13. RCT.tw.
14. random allocation.tw.
15. randomly allocated.tw.
16. allocated randomly.tw.
17. (allocated adj2 random).tw.
18. or/9-17
19. 8 and 18

Pubmed

1. “postpartum period”[MH]
2. postpartum OR post-partum OR postnatal OR post-natal OR puerperium OR postpartal OR post-partal OR lactating OR lactation OR “nursing women” OR breastfeeding OR breast-feeding OR “after birth” OR “following pregnancy” OR postpregnancy OR “post pregnancy” OR “following childbirth” OR “after delivery” OR “post childbirth”[TIAB]
3. 1 or 2
4. diet* [TIAB]
5. lifestyle AND (chang* OR intervention*) [TIAB]
6. physic* activ* OR exercis* [TIAB]
7. 4 or 5 or 6
8. 3 and 7
9. Clinical trial/
10. randomized controlled trials/
11. random allocation/
12. RCT.tw.
13. random allocation.tw.
14. randomly allocated.tw.
15. allocated randomly.tw.
16. or/9-15
17. 8 and 16

Limit: Humans

EBM reviews

1. Exp postpartum/
2. (postpartum OR post-partum OR postnatal OR post-natal OR puerperium OR postpartal OR post-partal OR lactating OR lactation OR "nursing women" OR breastfeeding OR breast-feeding OR "after birth" OR "following pregnancy" OR postpregnancy OR "post pregnancy" OR "following childbirth" OR "after delivery" OR "post childbirth").ti,ab.
3. 1 or 2
4. diet*.ti,ab.
5. (life*style ADJ2 (chang* OR intervention*)).ti,ab.
6. (physic* activ* OR exercis*).ti,ab.
7. 4 or 5 or 6
8. 3 and 7
9. Clinical trial/
10. randomized controlled trials/
11. random allocation/
12. randomi?ed controlled trial$.tw.
13. RCT.tw.
14. random allocation.tw.
15. randomly allocated.tw.
16. allocated randomly.tw.
17. (allocated adj2 random).tw.
18. or/9-17
19. 8 and 18

Cochrane Pregnancy and Childbirth Group.

1. MeSH descriptor: [Postpartum Period] explode all trees
2. (postpartum OR post-partum OR postnatal OR post-natal OR puerperium OR postpartal OR post-partal OR lactating OR lactation OR "nursing women" OR breastfeeding OR breast-feeding OR "after birth" OR "following pregnancy" OR postpregnancy OR "post pregnancy" OR "following childbirth" OR "after delivery" OR "post childbirth").(title, abs, keywords)
3. 1 or 2
4. diet*.(title, abs, keywords)
5. life*style AND (chang* OR intervention*) (title, abs, keywords)
6. (physic* activ* OR exercis*).(title, abs, keywords)
7. 4 or 5 or 6
8. 3 and 7

9. MeSH descriptor: [Clinical Trial] explode all trees
10. MeSH descriptor: [Randomized Controlled Trial] explode all trees
11. MeSH descriptor: [Random Allocation] explode all trees
12. randomized controlled trial (all text)
13. RCT (all text)
14. random allocation ((all text)
15. randomly allocated (all text)
16. allocated randomly (all text)
17. or/9-16
18. 8 and 17
19. #18 AND SR-PREG

Psycinfo

1. (postpartum OR post-partum OR postnatal OR post-natal OR puerperium OR postpartal OR post-partal OR lactating OR lactation OR “nursing women” OR breastfeeding OR breast-feeding OR “after birth” OR “following pregnancy” OR postpregnancy OR “post pregnancy” OR “following childbirth” OR “after delivery” OR “post childbirth”).ti,ab
2. 1 or 2
3. diet*.ti,ab.
4. (life*style ADJ2 (chang* OR intervention*)).ti,ab.
5. (“physic* activ*” OR exercis*).ti,ab.
6. 4 or 5 or 6
7. Clinical trial/
8. randomized controlled trials/
9. random allocation/
10. randomi?ed controlled trial$.tw.
11. RCT.tw.
12. random allocation.tw.
13. randomly allocated.tw.
14. allocated randomly.tw.
15. (allocated adj2 random).tw.
16. or/7-15
17. 6 and 16
18. Limit: Human

Embase

1. ‘puerperium’/exp

2. (postpartum OR post-partum OR postnatal OR post-natal OR puerperium OR postpartal OR post-partal OR lactating OR lactation OR “nursing women” OR breastfeeding OR breast-feeding OR “after birth” OR “following pregnancy” OR postpregnancy OR “post pregnancy” OR “following childbirth” OR “after delivery” OR “post childbirth”):ab,ti
3. 1 or 2
4. diet*:ab,ti
5. (life*style NEAR/2 (chang* OR intervention*)):ab,ti
6. (“physic* activ*” OR exercis*):ab,ti
7. 4 or 5 or 6
8. 3 and 7
9. Clinical trial/
10. randomized controlled trials/
11. random allocation/
12. randomi?ed controlled trial$.tw.
13. RCT.tw.
14. random allocation.tw.
15. randomly allocated.tw.
16. allocated randomly.tw.
17. (allocated adj2 random).tw.
18. or/9-17
19. 8 and 18
20. Limit: Humans

Table S2. Inclusion and exclusion criteria of the systematic review and meta-analysis of lifestyle intervention in postpartum women.

	Participants (P)	Intervention (I)	Comparison (C)	Outcomes (O)	Study type	Limits
**Inclusion Criteria**	Postpartum women (2 years post delivery).	Dietary, physical activity or behavioral interventions	Usual care, no intervention, or minimal intervention (single session at baseline)	Weight or weight change Total energy intake or change Physical activity or change	RCT	All languages, translation will be obtained whenever possible. Not limited by year.
**Exclusion Criteria**	Pregnant women	Allergen avoidance studies, acute studies, supplement trials Intervention that recruited during pregnancy Exercise intervention focusing only on pelvic floor exercise, urinary incontinence Intervention focusing only on initiating or increasing breastfeeding (without diet or exercise component)	Any dietary or physical activity intervention in the control arm that provides more contact than a single baseline information session.	Studies without relevant outcomes	Editorial, narrative review, conference abstract, letters, commentaries, uncontrolled trials, study protocol, non randomized controlled trials, studies with pregnant women will only be included if subgroup data is available for postpartum women	

Table S3. Definition of Health Literacy Lifestyle Intervention Domains.

Domain Name	Included Domains from HLQ	Example
Interacting with healthcare professionals	D6 -Ability to actively engage with healthcare providers (AE)	Example: a tool or intervention (practical) augments aids communication with HP<br>• Permission to ask questions<br>• Understanding roles of HPs<br>• Language to ask questions<br>• Understanding their own needs<br>• Reluctance of HPs to discuss weight/lifestyle refer to student's thesis Alexis
Access and utilisation of health care	D7 - Navigating the healthcare system (NHS)	Access and utilization of health resources. (knowledge and limitation around having a child) Teach to use existing resources in the community. Linkages.<br>Example: provide advice or resource list on where to seek help to manage weight and lifestyle, can include community resources
Self-care: skills and knowledge	D3 - Actively managing my health (AMH)<br>D2 - Having sufficient information to manage my health (HSI)<br>D5 - Appraisal of health information (CA)<br>D8 - Ability to find good health information (FHI)<br>D9 - Understand health information well enough to know what to do (UHI)	Example: goal setting, self-monitoring, meal or exercise plans, relapse coping, stress coping, cognitive behavioural therapy, specific time, intensity, duration of exercise<br>Example: Provide instruction on how to perform behaviour/diet/exercise, demonstration, onsite sessions
Social support or enabler	D4 - Social support for health (SS)	Example: social circle/peer, natural social circle including leveraging social relations enabling her health/purposeful interaction. Intentional/explicit/not-incidental. Social support as a resource to manage health.<br>Example: family based/partners<br>• Lack of intentional social support
Participating in health debates and decision-making	Not in HLQ	Example: Involve in the development of implementation of the intervention/co-design

Table S4. Risk of bias of included studies *.

Author	Randomisation Process	Deviations from Intended Interventions	Missing Outcome Data	Measurement of the Outcome	Selection of the Reported Result	Overall Bias
Berry 2015	Low	High	Low	High	Low	High
Bertz 2015	Low	High	Low	Low	Low	High
Colleran 2012	Low	High	Low	High	Low	High
Craigie 2011	Low	High	Low	Low	Low	High
Davenport 2011	Some concerns	High	Low	High	Low	High
deRosset 2013	Low	High	Some concerns	High	Low	High
Dritsa 2009	Some concerns	High	Some concerns	High	Low	High
Fjeldsoe 2010	Low	High	Low	High	Low	High
Holmes 2018	Low	High	High	High	Low	High
Huang 2011	Some concerns	High	Low	High	Low	High
Huseinovic 2016	Low	High	Low	High	Low	High
Keller 2014	Some concerns	High	High	Low	Low	High
Kernot 2019	Low	High	Some concerns	High	High	High
Khodabandeh 2017	Low	High	Low	High	Low	High
Krummel 2010	Some concerns	High	Low	High	Low	High
Leermakers 1998	Some concerns	High	Low	High	Low	High
Lioret 2012	Low	High	Low	High	Low	High
Lovelady 2000	Some concerns	High	High	Low	Low	High
Lovelady 1995	Some concerns	High	Low	Low	Low	High
Lovelady 2009	Some concerns	High	Low	High	Low	High
Maturi 2011	Low	High	Low	High	Low	High
McCrory 1999	Low	High	Low	High	Low	High
McIntyre 2012	Some concerns	High	Low	High	Low	High
Nicklas 2014	Low	Low	Low	Low	Low	Low

Ostbye 2009	Some concerns	High	Low	High	Low	High
O'Toole 2003	Low	High	Low	High	Low	High
Parsa 2017	Low	High	Low	High	Low	High
Tripette 2014	Some concerns	Some concerns	Low	High	Low	High
Wiltheiss 2013	Low	High	Some concerns	High	Low	High
Youngwanichsetha 2013	Low	High	Low	Low	Low	High
Zilberman 2018	Some concerns	High	Some concerns	Some concerns	High	High
Zourdalani 2015	Low	High	Low	Low	Low	High

* Based on the Revised Cochrane risk of bias tool for randomized trials (RoB 2.).

Table S5. Characteristics of included studies.

Study; Sample Size	Country	Postpartum Age; Postpartum Population	Intervention Type; Delivery Format	Intervention Provider; Intervention Location	Duration; Number of Sessions
Armstrong 2003<br>N = 20	Australia	6 weeks - 12 months postpartum<br>EPDS of ≥ 12	Exercise<br>Group	Dietitian<br>Outdoor and school hall	12 weeks<br>In-person: 36
Berry 2015<br>N = 60	USA	At least 6 weeks postpartum<br>BMI > 25 kg/m²	Diet and exercise<br>Group and individual	Health education interventionist<br>Health clinics and home	6 months<br>In-person: 15
Bertz 2015<br>N = 68	Sweden	10-14 weeks postpartum<br>Prepregnancy BMI 25 – 35 kg/m²	Diet and exercise<br>Individual	Dietitian and physical therapist<br>Clinic, home and outdoor	12 weeks<br>In-person: 4;<br>SMS: 24
Colleran 2012<br>N = 31	USA	4 weeks postpartum<br>BMI 25 to 30 kg/m²	Diet and exercise<br>Individual	Research assistants and dietitians<br>Home	16 weeks<br>Session number could not be determined
Craigie 2011<br>N = 52	UK	6 - 18 months postpartum<br>BMI > 25 kg/m²	Diet and exercise<br>Individual	A trained lifestyle counsellor<br>Home	12 weeks<br>In-person: 3;<br>Phone: 3
Daley 2015<br>N = 94	UK	Within 6 months postpartum<br>Depression according to	Exercise<br>Individual	Physical Activity Facilitator<br>Home	6 months<br>In-person: 2;<br>Phone: 2

		ICD-10 and EPDS			
Davenport 2011<br>N = 47	Canada	7 - 9 weeks postpartum<br>BMI ≥ 25.0 kg/m^2 and/or had retained ≥ 5.0 kg from pregnancy	Diet and exercise<br>Group and individual	Study investigator and registered dietitian<br>Centre and home	16 weeks<br>48-64 walking sessions
deRosset 2013<br>N = 24	USA	6 weeks postpartum<br>Overweight or obese by self-report according to prepregnancy BMI	Diet and exercise<br>Group	Not reported<br>Clinic, community and home	12 weeks<br>In-person: 12
Dritsa 2009<br>N = 88	Canada	4 - 38 weeks postpartum<br>EPDS ≥ 10	Exercise<br>Individual	Exercise physiologist<br>Home	12 weeks<br>In-person: 4
Fjeldsoe 2010<br>N = 88	Australia	Less than 12 months postpartum<br>General population	Exercise<br>Individual	Behavioural counsellor<br>Centre or home and community	12 weeks<br>In-person: 2;<br>SMS: 47 - 71
Haire-Joshu 2015<br>N = 1325	USA	Less than 1 year post-partum<br>General population	Diet and exercise<br>Group and individual	Parent educators<br>Home and school	12 months<br>In-person: 10 plus online
Holmes et al. 2018	USA	24 weeks postpartum	Edu program, weight management program,	Weight loss centre<br>Phone/text	3 months

		Postnatal overweight with PH of GDM	weight monitoring, tele and text support.		
Huang 2009<br>N = 240	Taiwan	24-48 h - 6 months postpartum<br>General population	Diet and exercise<br>Individual	Nurse<br>Hospital and clinic	6 months<br>In-person: 3
Huseinovic 2016<br>N = 110	Sweden	6 - 15 weeks postpartum<br>BMI ≥ 27 kg/m^2	Diet<br>Individual	Dietitians<br>Home	12 weeks<br>In-person: 1;<br>Text message: 12; Phone: 12
Keller 2014<br>N = 139	USA	6 weeks - less than 6 months postpartum<br>BMI ≥ 25 kg/m^2	Exercise<br>Group	Community health worker<br>Outdoor	12 months<br>In-person: 52
Kernot et al. 2019	Aust	6 week- 6 month postpartum<br>Postpartum (facebook)	Exercise<br>Group (virtual)	Electronic messaging via app<br>Home	6 weeks<br>Weekly emails
Khodabandeh 2017<br>N = 220	Iran	Day of discharge postpartum<br>General population	Diet and exercise<br>Individual	Researcher<br>Hospital and home	6 weeks<br>In-person: 2;<br>Text message: ~ 8
Krummel 2010<br>N = 151	USA	Up to 2 years postpartum<br>General population	Diet and exercise<br>Group and individual	Trained facilitators<br>Centre and home	12 months<br>In-person: 11
Lee 2016<br>N = 65	UK	6 weeks - 1 year postpartum	Exercise	Health psychologist<br>Local university or home	3 months<br>In-person: 12

		General population	Group and individual		
Leermakers 1998<br>N = 90	USA	3 - 12 months postpartum<br>Exceed their pre-pregnancy weight by at least 6.8 kg	Diet and exercise<br>Group and individual	Not reported<br>Centre and home	6 months<br>In-person: 2;<br>Phone: 12-24
Lioret 2012<br>N = 542	Australia	18 months postpartum<br>General population	Diet and exercise<br>Group	Dietitian<br>Health centres	15 months<br>In-person: 6
Lovelady 2000<br>N = 48	USA	4 weeks postpartum<br>BMI 25 to 30 kg/m^2	Diet and exercise<br>Individual	Trained research assistants<br>Home and study centre	10 weeks<br>In-person: 40
Lovelady 1995<br>N = 38	USA	6 weeks postpartum<br>General population	Exercise<br>Individual	Not reported<br>Centre-based	12 weeks<br>In-person: 60
Lovelady 2009<br>N = 24	USA	3 weeks postpartum<br>BMI 20 to 30 kg/m^2	Exercise<br>Individual	Research Assistants<br>Home	16 weeks<br>In-person: 48
Maturi 2011<br>N = 70	Iran	6 weeks - 6 months postpartum<br>BMI > 19.8 and < 29 kg/m^2	Exercise<br>Individual	Researchers<br>Home and community	12 weeks<br>In-person: 1;<br>Text message: 12; Phone: 6
McCrory 1999<br>N = 68	USA	12 ± 4 weeks postpartum	Diet and exercise<br>Individual	Not reported<br>Location not reported	11 days

		General population			
McIntyre 2012<br>N = 28	Australia	6 weeks postpartum<br>Post gestational diabetes	Exercise<br>Individual	Exercise physiologist<br>Home	12 weeks<br>In-person: 1;<br>Phone: 8
Nicklas 2014<br>N = 75	USA	6 weeks postpartum<br>Post gestational diabetes	Diet and exercise<br>Individual	Licensed dietitian<br>Home	12 months<br>Cannot be determined
Ostbye 2009<br>N = 450	USA	6 weeks postpartum<br>Pre-pregnancy BMI ≥ 25 kg/m²	Diet and exercise<br>Group and individual	Facilitators; Counsellor<br>Community and home	9 months<br>In-person: 18<br>Phone: 6
O'Toole 2003<br>N = 40	USA	6 weeks - 6 months postpartum<br>Pre-pregnancy BMI 25-29.9 kg/m²	Diet and exercise<br>Group and individual	Dietitian and exercise physiologist<br>Research lab and home	Cannot be determined
Parsa 2017<br>N = 120	Iran	3 - 20 days postpartum<br>General population	Diet and exercise<br>Group	Not reported<br>Health centres	3 weeks<br>In-person: 3
Tripette 2014<br>N = 34	Japan	3 months - 1 year postpartum<br>BMI > 22 kg/m²	Exercise<br>Individual	Wii Fit Plus game<br>Home	40 days<br>In-person: 2
Wiltheiss 2012<br>N = 400	USA	Within 6 months postpartum<br>BMI ≥ 25 kg/m²	Diet and exercise<br>Group and individual	Trained telephone counsellors and study nutritionists<br>Home and study centre	8 months<br>In-person: 1;<br>Mail: 8;<br>Phone: 8

Youngwanichsetha 2013<br>N = 69	Thailand	6 -12 weeks postpartum<br>Type 2 diabetes	Exercise<br>Individual	Not reported<br>Hospital for training and home for exercise	12 weeks<br>In-person: 3
Zourladani 2015<br>N = 42	Greece	4 - 6 weeks postpartum<br>General population	Exercise<br>Group	Fitness instructor<br>Centre	12 weeks<br>In-person: 36
Zilberman et al. 2018	Israel	3-4 months postpartum<br>General population	Lifestyle (diet/exercise)<br>Individual	Dietitian/sports instructor<br>Gym	24 months<br>3 individual sessions<br>4 group

Table S6. Health literacy domains of lifestyle interventions in postpartum women.

Author	Interacting with Healthcare Professionals	Access and Utilisation of Health Care	Self-care: Skills and Knowledge	Social Support or Enabler	Participating in Health Debates and Decision-making
Berry 2015			X	X	
Bertz 2015			X		
Colleran 2012			X		
Craigie 2011			X		
Daley 2015	X				
Davenport 2011			X		
deRosset 2013			X		
Dritsa 2009			X		
Fjeldsoe 2010			X	X	
Holmes 2018			X		
Huang 2011			X		
Huseinovic 2016			X		
Keller 2014	X		X	X	
Kernot 2019			X		
Khodabandeh 2017			X		
Krummel 2010			X	X	
Leermakers 1998			X	X	
Lioret 2012			X	X	
Lovelady 2000			X		
Lovelady 1995			X		
Lovelady 2009			X		
Maturi 2011			X		

McCrory 1999			X		
McIntyre 2012			X		
Nicklas 2014			X		
Ostbye 2009	X		X	X	
O'Toole 2003			X	X	
Parsa 2017			X	X	
Tripette 2014			X		
Wiltheiss 2013			X	X	
Youngwanichsetha 2013			X		
Zilberman 2018			X		
Zourdalani 2015			X		
